# Implementing team‐based learning in the life sciences: A case study in an online introductory level evolution and biodiversity course

**DOI:** 10.1002/ece3.6863

**Published:** 2020-12-17

**Authors:** Lina M. Arcila Hernández, Kelly R. Zamudio, Abby G. Drake, Michelle K. Smith

**Affiliations:** ^1^ Department of Ecology and Evolutionary Biology Cornell University Ithaca NY USA

**Keywords:** distance education, evolution & biodiversity education, online learning, remote learning, team‐based learning

## Abstract

Team‐Based Learning (TBL) is a pedagogical tool that has great potential to develop student engagement, accountability, and equity in the online classroom. TBL is rooted in evidence‐based educational theories and practices that underlie many active learning approaches such as self‐testing, team discussion, and application of knowledge. The use of these approaches is associated with better student performance, retention, and sense of belonging in the classroom, aspects that are often reported to be especially lacking in online courses. Here, we describe how we implemented TBL in a face‐to‐face and an online introductory level evolution and biodiversity course. We implemented TBL in the face‐to‐face course (~200 students) starting in 2018 and in the online course (~30 students) starting in the summer of 2019. We used several online applications to facilitate the transition to an online platform such as Simbio, Slack, VoiceThread, Articulate 360, and Teammates. Our experiences using TBL approaches in the online course have been rewarding, and students are engaged and accountable for their learning and performed well in the course. Our goal is to provide an example of how we designed a life science course using TBL approaches and transitioned the course to an online environment. With the current switch to remote instruction and online learning, we recommend the use of TBL as a course design approach that can improve the students’ online learning experience.

## Graphical abstract

Implementing Team‐based Learning (TBL) strategies and teamwork in online life science courses improves student engagement, equity, and accountability. Here, we provide a case study demonstrating how to use TBL in an online evolution and biodiversity course. With the current switch to remote instruction and online learning, we recommend the use of TBL as a course design approach that can improve the students’ online learning experience.

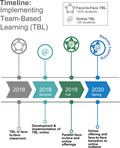

## 1 INTRODUCTION

Online learning has been on the rise in degree‐granting universities over the last decade (Seaman et al., 2018) and describes courses thoughtfully designed to deliver learning materials and support students attending the courses mostly or fully online, either asynchronously or synchronously (Hodges et al., 2020; Means et al., 2014; Seaman et al., 2018). A benefit of online learning is that it allows for higher student enrollment, with students having a higher degree of place and time flexibility to take these classes. All of these factors are especially relevant for students who are in learning abroad programs, are athletes, have disabilities, or are working (Branch & Dousay, 2015; Means et al., 2014).

The flexibility of offering online course options became critical with the need for social distancing during the COVID‐19 pandemic, as suggested by the number of countries that initially moved to complete or partial online teaching (Crawford et al., 2020) and the increase in online learning searches on internet (Lashley et al., 2020). In March 2020, students in North America, and many around the world, returned to their places of residence and transitioned to remote instruction to complete courses in progress (Crawford et al., 2020; Zhang et al., 2020). Because this was a temporary fix to disruption of face‐to‐face courses and instructors did not often have time or training to design a thoughtful online course, this form of instruction is often described as remote instruction (Hodges et al., 2020). Remote instruction recapitulates the face‐to‐face course but does not necessarily implement online course design elements to facilitate learning (Hodges et al., 2020; Means et al., 2014).

While remote instruction during COVID‐19 was necessary, it has limitations. One of the main limitations is inherent to remote instruction, during an emergency few instructors had the time to thoroughly consider or implement online tools designed to improve learning in an online environment. Careful course design is especially important because student retention rates are low in online offerings—a trend that is usually linked to a lack of student engagement, accountability, and sense of belonging within the class (O’Keeffe, 2013; Zhu et al., 2020). Instructors of remote instruction were also adjusting to having fewer opportunities for immediate feedback to help resolve students’ misunderstandings which imposes barriers to achieve positive learning outcomes (Clark et al., 2018; Kim et al., 2005). Currently, 23% of over 1,000 universities in the USA are planning to switch to hybrid or fully online courses during the Fall term of 2020 (Chronicle Staff, 2020). To maintain the quality of our teaching, it is essential that these courses move beyond remote instruction to use online course design tools that provide adequate learning support to undergraduates in the online classroom (Branch & Dousay, 2015; Means et al., 2014).

**Figure 1:**
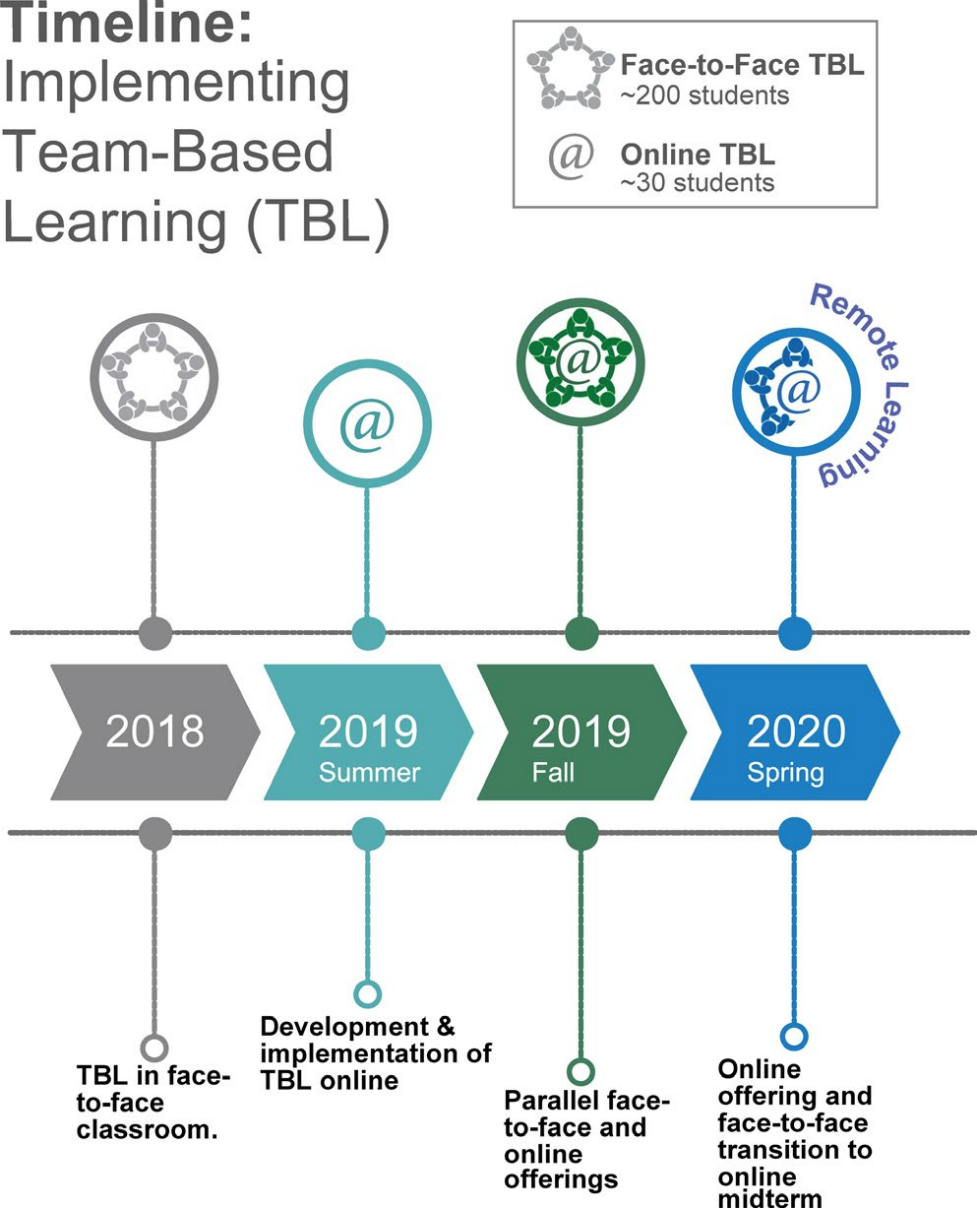
Timeline of TBL implementation in an introductory level evolution and biodiversity course

Prior to the COVID‐19 pandemic, we worked on addressing some of the remote instruction and online learning pitfalls by purposefully designing an online introductory level evolution and biodiversity course using a Team‐Based Learning (TBL) approach. When remote instruction was mandated in March 2020, we were undergoing our third term teaching the online course in parallel to the face‐to‐face course (See timeline in Figure 1), and we scaled up our efforts to accommodate all face‐to‐face students in our online platform for the remainder of the semester. Here, we discuss our successful use of TBL in a large face‐to‐face introductory life science course and how we adapted it to the online platform. We describe pedagogical and technical tools used, as well as the perceived challenges and benefits of implementing TBL in an online course. We also provide a summary of helpful practices and useful literature for those interested in implementing team‐based approaches in an online platform.

## 2 TEAM‐BASED LEARNING: A LIFE SCIENCE FACE‐TO‐FACE CASE STUDY

When recalling what a large introductory life science course looked like during our time as undergraduates, most of us would agree that it could be intimidating and lonely. Often it was an instructive but lackluster experience where a myriad of information was conveyed by a lecturer. Clearly, some students were fortunate to have incredible lecturers, but these exceptions were not the norm. A national report assessing course strategies in STEM (science, technology, engineering, and mathematics) fields showed that most STEM courses use lectures as their main teaching strategy with few courses using student‐centered strategies (Stains et al., 2018).

Compared to traditional introductory life science courses which place the lecturer in the center of the classroom, TBL classrooms are centered around the student. While TBL uses techniques (e.g., informational videos before lectures) associated with other student‐centered practices such as flipped classrooms, it is pedagogically distinct (Nishigawa et al., 2017). Unlike flipped classrooms, TBL uses collaborative learning theory and creates accountability through structured individual and team assessments (Michaelsen & Sweet, 2008; Nishigawa et al., 2017). TBL courses prepare students before entering the classroom by requiring them to do pre‐class assignments, increase accountability by engaging students in individual and team quizzes that reinforce pre‐lecture materials, and help students learn by frequently using polling and team activities during class (Michaelsen & Sweet, 2008). As a result, students are often engaged in lively conversations about core concepts in a TBL classroom. This shift from a lecture‐focused to student‐centered classroom improves students’ understanding of the learning objectives and ability to apply concepts beyond the end of the course (Armbruster et al., 2009; Tanner, 2013). The use of active learning activities improves overall student performance and retention, in addition to providing equitable opportunities to under‐represented minority students in STEM (Ballen et al., 2017; Freeman et al., 2014; Theobald et al., 2020). Therefore, TBL provides a framework to achieve student engagement, desired learning outcomes, and retention in the classroom (Clark et al., 2008, 2018; Michaelsen & Sweet, 2008).

**Figure 2:**
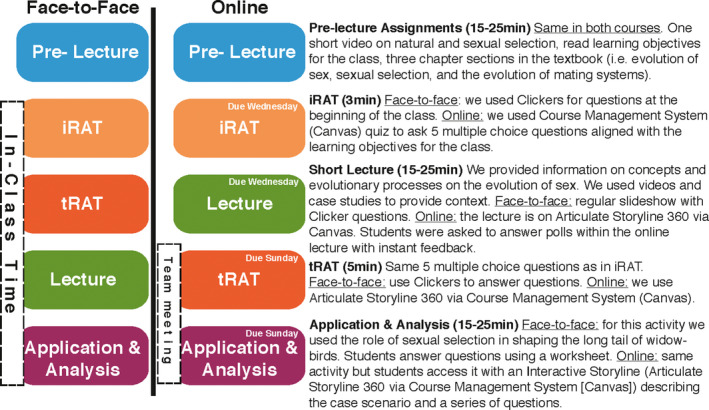
Example of TBL in a Face‐to‐Face and online lecture on Sexual Selection. Notice that the order of tRATs and Lectures are swapped in the online offering compared to the face‐to‐face offering to facilitate synchronous team meetings

We implemented TBL in the face‐to‐face introduction to evolution and biodiversity course at Cornell University, a class with an average enrollment of 200 students. The students are placed in formal pre‐determined teams of five students for the duration of the term. Formal teams provide students with peers to discuss course material and create interdependence among team members, promoting community and accountability in a big classroom (Donovan et al., 2018; Michaelsen & Sweet, 2008). Several team building practices can be used to create strong and cohesive teams, most of them requiring careful consideration of several demographic and academic variables (Donovan et al., 2018). We created teams that were diverse in gender and other intersectional identities (science education, nationality, majors, etc.), by asking students to fill out a short survey about several of these identities prior to the first week of classes (see survey on Appendix 1). Students come prepared to class to take an individual quiz (Individual Readiness Assurance Test [iRAT]; Michaelsen & Sweet, 2008) based on the pre‐lecture assignments (e.g., short videos and readings), then they revisit and clarify the materials a second time by taking the same quiz with their teammates (Team Readiness Assurance Test [tRAT]; Michaelsen & Sweet, 2008). After a short lecture, the students work together on applying concepts they learned about to real‐life scientific scenarios (See Figure 2 for a class structure example). Why is this extremely structured course so popular with some instructors and students? The short answer: TBL has strong positive outcomes for students; not only do students get higher scores and understand concepts better, but they also experience increases in accountability, sense of belonging, and retention (Ballen et al., 2017; Donovan et al., 2018; Kim et al., 2005; Michaelsen & Sweet, 2008).

## 3 IMPLEMENTING TBL IN AN ONLINE LIFE SCIENCE COURSE

With our success using TBL in the evolution and biodiversity face‐to‐face course, we transferred materials to an online platform. We wanted to offer this online course opportunity for students that had schedule conflicts or that were studying abroad. Our initial online offering in the summer of 2019 was opened for up to 30 students with the vision of expanding to a larger course of 100 or more students. Thus, we were able to successfully scale up when switching the large face‐to‐face offering to online learning during the required social distancing period in the spring of 2020. We preserved all of the learning outcomes used in the face‐to‐face course and also made high‐quality storylines, described below, appropriate for online delivery via the Canvas Course Management System. We pre‐assigned teams, with four students per team, for the term using the same team building guidelines described in the face‐to‐face offering. We added two questions to the team survey about time zones and preferred work times to facilitate synchronous meetings among team members (see survey questions on Appendix 1). We also modified the face‐to‐face TBL structure by moving the tRATs after the lecture, to increase schedule flexibility by minimizing the number of team synchronous meetings each week (See Figure 2). Individual assignments (pre‐lecture quizzes, iRATs, and lectures) were due mid‐week and included pre‐lecture and lecture materials. Sections and all team assignments associated with a lecture, including tRATs and Application & Analysis, were due after lecture on the weekend. Our goal was to create an engaging and inclusive course where students were accountable for their efforts.

### 3.1 Strategies used to enhance student accountability and provide immediate feedback

We used different applications and online tools throughout the course to enhance student accountability and course engagement (see Table 1). The course was organized by weeks (16 weeks total during a regular term), each week had two asynchronous lectures and one section activity (i.e., worksheet). At least one of the lectures each week implemented the entire TBL structure (iRAT, tRAT, and Application & Analysis) while other lectures only included the Application & Analysis component. We felt this was important so as not to overwhelm the students with multiple team quizzes per lecture. Figure 2 shows how we implemented these components in an asynchronous online environment.

**Table 1:** TBL elements and tools used to implement them in an online environment

TBL and Course elements	Pedagogical value*	Student development*	Tools used in this online case study	Alternative tools
Pre‐lecture	Preparation, Acquiring unit content	Individual accountability, Self‐efficacy	Videos: Panopto.com and YouTube.com^a^ video captioning; online textbooks	QuickTime Player^a^, Zoom recording^a^
iRAT, or pre‐lecture quiz	Retrieval, Practicing content	Individual accountability, Self‐efficacy	Course Management System (Canvas) Quiz	https://articulate.com/360 https://polleverywhere.com ^a^
tRAT	Retrieval, Practicing content, Immediate feedback	Team building and accountability, Self‐efficacy, Sense of belonging	Answer until correct quiz: https://articulate.com/360	https://polleverywhere.com ^a^
Lecture	Review content, Knowledge integration, Immediate feedback	Individual accountability	Slideshow with polling questions: https://articulate.com/360; General discussions: Slack.com^a^	Course Management System discussion boards
Team Application & Analysis	Knowledge integration, Decision making, Application and analysis of unit content, Immediate feedback	Team building and accountability, Sense of belonging, Self‐efficacy	Slideshow and Answer until correct quiz: https://articulate.com/360; Slideshow Discussions: https://voicethread.com. Team feedback: https://teammatesv4.appspot.com/ ^a^	Course Management System discussion boards
Sections	Knowledge integration, Decision making, Application, and analysis of unit content	Individual accountability, Self‐efficacy	Worksheets; Simulations: https://simbio.com	https://biointeractive.org;^a^ http://virtualbiologylab.org;^a^ and many others online
Tests	Long‐term retrieval and application	Individual accountability, Self‐efficacy	Face‐to‐Face testing; online open book testing	Online Proctoring services

Note

We list the resources we used or alternative tools that we considered as a starting point for instructors. With this list, we are not commenting on the efficacy of these tools and we acknowledge that other tools might be used for similar purposes (*Pedagogical value and student development are inferred based on Ambrose et al., 2010; Michaelsen & Sweet, 2008).

^a^ Free or open source applications.

We continued to use pre‐lecture individual assignments in the online course (Figure 2). These assignments included readings from an online textbook, short video lectures (5–15 min) describing basic concepts and processes, and a pre‐lecture quiz (or an iRAT if the lecture implemented the TBL structure). Regular pre‐lecture quizzes did not have a time limit, and students had two attempts to answer the quiz. Five questions were randomly selected from a quiz bank each time (quiz banks with 10–30 questions were populated using questions from previous tests). The students had immediate feedback on the answers and tips on how to answer the questions correctly if necessary. Alternatively, iRATs had a time limit of three minutes where the students answered five multiple‐choice questions. The students only had one chance to answer the iRAT questions and they had no immediate feedback on the answers because they would revisit those questions with their team during the tRAT.

Students could access lectures any time after having completed the pre‐lecture assignments. Lectures were similar to a slide show used in face‐to‐face classrooms, and students would open a lecture to browse through an interactive slideshow. In each lecture, we included a combination of images, text, animations, closed‐caption videos, voice over slides, and embedded multiple‐choice questions that were the same as the clicker questions we use in the face‐to‐face class. We developed these interactive lectures (i.e., interactive storylines) using Articulate Storyline 360 and embedded them in our course management system (Canvas) as an external tool. Making the lectures an external tool allowed us to score the questions embedded in the lectures and provided the students with immediate feedback on each question. Students could only answer the questions once. Question feedback was provided by using several slides explaining the correct answer and a few reasons of why the other answers to the question were incorrect. These interactive storylines gave us the tools to address students’ misunderstandings at an early stage.

### 3.2 Activities and strategies used to engage students in team discussions and analysis of the concepts learned

Post‐lecture assignments consisted of team activities that allowed students to apply the concepts learned. We also used Articulate Storyline 360 to implement these team assignments which included tRATs and Team Application & Analysis. With this tool, the students had the option to answer until correct whenever they encountered a multiple‐choice question in the team assignments. We also developed other team activities where we wanted students to expand on their discussion and have a record of the students reasoning. For those activities, we used VoiceThread (https://voicethread.com), an online tool that allows for asynchronous commentary through text, audio, or video recordings on a set of slides. We asked all students to post their first comment by Wednesday and then to build the discussion by replying to another team member comment after that. By the end of the week, all students had at least one comment on each slide, and the team had an agreement on their final answer and posted the final answer. In the assignment instructions, we emphasized that we only grade the final answer for correctness but participation points are awarded individually depending on engagement.

Finally, most section activities were individual worksheets with problem sets. Phylogeny building and interpretation were required in several case studies exploring issues related to health, biodiversity, and conservation throughout the term. Application of population genetics and natural selection modules relied on students’ interpretations of simulations or conservation case studies. Students were allowed to discuss these exercises with their teams but each student was required to submit their own worksheet; except for one section activity where the students used VoiceThread to discuss a primary literature paper with their team, followed by an individual Canvas quiz. We also used virtual tours to familiarize students with the university's entomological collection and plant conservatory. All of these activities were developed to revise previous course content and provide a space for the students to learn about how evolution and biodiversity principles are used beyond the classroom.

### 3.3 Increasing student engagement and accountability through team structure, peer‐to‐peer feedback, and discussion boards

Students were held accountable for their participation in the course and their contributions to the team in several ways. iRATs and within‐lecture questions ensured that students were individually responsible for their own understanding of the course material. Each team had a team leader whose role was to organize online meeting schedules and was also the person in charge of submitting the assignments. The team leader role rotated weekly among the students, and by the end of the term, each student took on the leadership role three or four times.

In addition, students within a team provided each other with anonymous peer‐to‐peer feedback by completing peer evaluations in Week 5. We asked students to provide this anonymous feedback to their team members using the TEAMMATES website (https://teammatesv4.appspot.com/). Our peer evaluations consisted of 10 multiple‐choice questions (answer choices: always, often, sometimes, never) such as:

- Our team functions well because this person is well prepared for team activities.
- Our team functions well because this person makes sure everyone on the team has a chance to speak and is heard.

They also wrote one or two sentences for each team member about:

- What is the single most valuable contribution this person makes to your team?
- What is the single most important way this person could change their behavior to more effectively help your team succeed?

Instructors reviewed the answers before sending them to the students. These evaluations were essential in positively modifying student behavior as peer feedback has a strong impact on students. We often saw students that were not engaged prior to peer evaluations become more responsible and involved after evaluations.

To facilitate teamwork and discussions, we created a Slack workspace with a general channel for the class. Each team also had their own channel to organize logistics, discuss lectures, and team assignments. Slack has the advantage that it can be used in multiple platforms (i.e., phones, computers, tablets, etc.), and it has been developed to facilitate team communication and productivity. Students gained participation points for actively engaging with other students on Slack. All students were invited to participate in the Slack workspace during the first week of classes. Once the students accepted the invitation, they were automatically added to the class general channel where the instructor answered commonly asked questions, provided logistical information to the class, and included current events and news related to course content. Students were encouraged to ask questions about lectures and problem sets in this general channel. Each student was also added to a private channel with their team members. The instructor introduced all team members and provided an icebreaker activity to motivate discussion in the team channel. Students were encouraged to use the team channel to get in contact with each other, schedule meetings, ask questions about lectures and sections, and to discuss questions posted by the instructor on relevant topics. Every week, we had students sending direct messages to contact the instructors and active participation within team channels. The general channel was often used by students when tests were getting closer to ask about test logistics and clarifying course topics.

### 3.4 Summative assessments

For both courses, students took three tests during the term and a final test. Whenever possible we had the online, students take these tests in person. However, when it became necessary to provide remote online assessments, we chose to have open book problem sets. We generated question banks for each lecture in Canvas and set up a test with question groups that selected one or two questions per lecture. Students had access to their individualized problem set for 24 hr.

### 3.5 Instructor‐to‐student ratio and grading

We taught the online course under different instructor‐to‐student ratios. We successfully taught the online course with one instructor for about 30 students, and the number of assignments proposed here works best if the instructor has teaching support from one teaching assistant for every 20–25 students. If one instructor per every 20–25 students is not possible, we suggest increasing the number of team and section activities that are auto‐graded (i.e., using multiple‐choice poll questions or quizzes). Alternatively, the instructors can also create peer‐review assignments or have one or two main projects instead of weekly section worksheets, which are graded individually.

## 4 MOVING A LARGE FACE‐TO‐FACE LIFE SCIENCE COURSE ONLINE

In March 2020, our face‐to‐face class of 170 students transitioned to online teaching as our campus was shutdown. We were fortunate that we had a fully developed online course already available to us. We cloned the online course that we had developed in our Course Management System (Canvas) and enrolled all face‐to‐face students in this new online Canvas site. We recorded instructional videos for the students about how to navigate the online course and held several Canvas Conference online meetings for the students to ask the instructors questions. On this new online platform, the students continued to work on assignments both individually and with their teams (team members remained the same as in the face‐to‐face course). Instructors that were originally scheduled to lecture in the face‐to‐face course held online question and answer sessions twice a week to help students with content. Our discussion sections were now asynchronous assignments, so our 7 graduate teaching assistants and our 5 undergraduate teaching assistants were able to offer nearly 40 hr a week of online office hours. Largely because we had an online course already developed our students did much better than we expected. We originally thought that many would drop the course or fail. Only two students dropped after we transitioned online, all students passed, and two students took incompletes.

## 5 BENEFITS OF IMPLEMENTING TBL IN ONLINE PLATFORMS

### 5.1 Student accessibility and equity

Online learning allows for schedule flexibility and the use of multiple tools that allow for different modes of knowledge representation and assessments. While our course requires nearly 16 hr of work per week, as expected of 4–5 credit courses, students have the flexibility to go over the different assignments at their own pace and time of choosing. Students also determine their best time to meet as a team each week. Research shows that in large courses, course pace is a significant concern for students (Meaders et al., 2020). In contrast, students often commented in our online course that self‐pacing and taking the time necessary for them to complete a lecture was one of the strengths of the class, highlighting the importance of asynchronous options in online course design.

We also implemented Universal Design of Learning (UDL) and Digital Accessibility practices in our course. UDL encourages the use of multiple modes of engagement, representation, and expression to support inclusivity and student diversity in the classroom (Rose et al., 2006). Meanwhile, Digital Accessibility practices permit any student, regardless of disability status, to access all of the information available in the course (EOWG, 2019). Video captioning and Alternative Text for figures are examples of online accessibility practices (see more in EOWG, 2019). Together these two approaches allow for accessibility in a variety of assignments and reinforcement of concepts under different contexts, and increased equity in our course. Students that use text‐to‐speech readers, that need more time with the materials, international students with First Language not English (FLNE) or students that cannot commute to campus can access all of the materials in a way that is engaging and practical. Student surveys showed that pre‐lecture videos and associated video transcripts are one of our students’ favorite learning tools.

Finally, rotating team leader roles and providing multiple discussion venues assured that all student voices were heard throughout the term. Rotating roles assures that leadership is equally shared among all the students. It also allows practice for students that are less comfortable in those positions and that would not volunteer to participate otherwise, thus encouraging for more equitable participation of all team members regardless of gender, race, or experience (Aguillon et al., 2020; Ballen et al., 2019; Tanner, 2013).

### 5.2 Student accountability

To increase accountability, each class component (i.e., pre‐lecture assignments, lectures, iRATs, tRATs, and application & analysis) was set up in a required progression, where students needed to complete the previous step before they could continue to the next assignment. For example, students had to review all of the pre‐lecture assignments before the iRAT or lecture would unlock. Similarly, students could only access the team assignments if they had completed the individual work. Although students commented that the large number of small assignments was challenging, they rarely missed one of these deadlines and performed well on them. Students also commented that having the opportunity to earn points throughout the course was more desirable than having their grades depend mostly on a few high‐stakes tests.

We also found that most teams presented actions that can be linked to team cohesion, with all members participating weekly in meetings and submitting team assignments on time. To assure participation in team activities, we asked teams to upload photographs or screenshots of their online meetings with all team members present. Furthermore, a voluntary mid‐semester team feedback survey suggested that most students were prepared to work on team assignments. Students often checked‐in with each other on discussion boards about meeting times and final answers for team assignments. Overall, students commented on how the structure of the course encouraged them to not fall behind on assignments and provided them with opportunities to clarify concepts with team members.

## 6 CHALLENGES OF IMPLEMENTING TBL IN ONLINE PLATFORMS

### 6.1 Team set up and scheduling

Although most teams worked together successfully, we encountered two major challenges when trying to set up teams and maintaining communication among team members. First, we observed that long enrollment and course drop periods were detrimental when trying to establish team rapport early in the semester. However, many students used the enrollment period to determine whether they were to stay in a course or not, creating a lot of flux in the student roster and any teams established during this time. Furthermore, those students that were committed to the class but that were part of a team with a changing membership seemed unlikely to create strong team rapport later in the term. We opted to create teams with at least four members to allow for students dropping the class during the first three weeks of the term. Second, several teams had challenges communicating or finding a time to schedule weekly meetings. This was especially problematic for teams when students had full‐ or part‐time jobs in addition to a full course‐load. We found that determining weekly availability when building teams and proving scheduling tools (e.g., online polls) were essential for the long‐term success of the team. Going forward, we are requiring the teams to determine two, hour long, weekly meeting times that they will commit to for the whole term.

### 6.2 Methodological and technical challenges

Our class was developed using a Universal Designed for Learning, providing learning opportunities for students across different media and activities. We used several online technologies to implement such activities. However, the use of multiple tools increases the likelihood of methodological and technical issues, and some of the tools can also be costly. Our first challenge was to familiarize the students with all the tools without making the course overwhelming. Students found the first week of our course confusing given the large number of small assignments. There was a new course structure to learn in addition to several online tools. We provided detailed instructor guidance during the first two weeks of the course and explained how the different assignments translated to the different components of a face‐to‐face course. We also provided different venues to reach out to instructors: weekly online office hours, email, and Slack messaging. We also sent reminders on the days when assignments were due and checked‐in on students frequently to ensure they were working on their tasks. In a large class, this can be overwhelming for one instructor so we enlist the help of our graduate student TAs in tracking student progress.

Our second challenge was to synchronize all of the platforms to work seamlessly. Canvas Course Management System and other similar platforms provide the ability to use external tools to engage the students. However, some of the external tools do not necessarily interface with Canvas seamlessly. We had several issues with internet browsers disabling score transfers from external tools to Canvas gradebook, software upgrades removing important features for our assignments, or lack of features in Canvas to allow easy use of the team option with an external tool. Instructors should be aware that a portion of the teaching time will be used troubleshooting and minimizing these technological issues.

## 7 PROMISING PRACTICES

- Explore TBL online resources. Before developing your course, we recommend reading or visiting the following resources: Clark et al., 2018 reviews best practices for implementing TBL online. Palsolé and Awalt (2008) describes a different TBL case study with asynchronous team discussions. Clark and Leonard (2016; https://sites.google.com/site/tbladvantageschallenges/welcome‐video) provides an example of an online module and further information on TBL practices.
- Survey students before creating teams. Team building is perhaps one of the most essential steps to successfully use TBL online. A short 1‐ to 5‐min survey can facilitate this task. One of the first questions should be time availability to work with teams during the week or weekend. After asking about time availability and the time zone for each student, different methods can be used to group the students (several methods summarized in Donovan et al., 2018). We suggest considering questions regarding gender, under‐represented status, and other relevant experiences for team work (e.g., playing instruments or student membership in a varsity team) (Woolley et al., 2015).
- Share tools and skills that students can use when working in teams. Most students had never worked in online teams before the social distancing restrictions. It is important to show them tools and behaviors that can facilitate team communication. An instructor could share online applications such as When2meet or doodle poll, demonstrate how to set up recurrent meetings, and model respectful online discussion behaviors.
- Provide strategies and expectations for good teamwork. The instructor should guide the students understanding of team rules. For example, ask the students to discuss what would happen if one team member does not show up for a meeting on time, would the team wait for five minutes before starting the meeting or delay the meeting?
- Provide an estimated time to completion for every assignment. Students often ask how often they need to meet and for how long. It is easy to add an estimate of time to the assignments description and it allows the students to schedule their weekly team meetings more efficiently.
- Use participation points in online discussions. In our experience, providing incentive for discussion boards often results in a more active discussion among team members and helps generate accountability (Aloni & Harrington, 2018). We also find that asking for photographs or transcripts of online meetings helps assure that all students are participating during online synchronous meetings.
- Maintain active instructor and student interactions. There is some evidence that a higher number of interactions between instructors and students help generate engagement and cohesion in the online classroom (Chatterjee & Correia 2020). Instructor–student interactions are particularly relevant at the beginning of the course when students are learning how to engage with the course and team members. We recommend that instructors initiate daily interactions through discussion boards and announcements during the first one or two weeks of classes, as well as answering emails and messages promptly.
- Use of multiple tools for discussions. Different discussion platforms provide opportunities for the students to engage with the material more thoroughly. We found that structured discussions such as those using VoiceThread were useful to enhance student participation, practice recalling concept, and applying those concepts to new scenarios. Other discussion platforms, such as Slack or other discussion boards, allowed for spontaneous discussions with students building up on each other ideas and creating informal conversations that might enhance the sense of belonging in the class.

## 8 CONCLUSION

Overall, TBL enhanced the student experience in our online course. Our experience showed not only that students participated actively in the course but also acquired high performance levels. This outcome was especially demonstrated when we transitioned our face‐to‐face class to online teaching in the Spring of 2020. To our surprise, all of our students not only completed our course (despite the opportunity to withdraw without penalty) but also did very well—only two students had final grades below 70% and none failed the course. In addition, discussions and assessments demonstrated that students had a clear understanding of fundamental evolutionary topics.

Students reported the usefulness of teamwork but found scheduling meeting times a challenge and an added stressor, which underscores previous research on online courses with team work components (Goh & Gunnells, 2020; Palsolé & Awalt, 2008). We recommend requiring teams to set weekly meeting times at the start of the term. Other online courses that implemented TBL opted for only asynchronous team discussions (e.g., Palsolé & Awalt, 2008). However, we found that synchronous team discussions in our online course had components usually associated with enhanced sense of belonging and accountability. We suggest that online courses continue assessing the role of synchronous and asynchronous discussions in different student populations. Overall, our experience transitioning to online learning during the pandemic confirmed that the use of TBL strategies and teamwork can help increase student engagement, equity, and accountability in online life science courses.

## CONFLICT OF INTEREST

The authors declare no conflict of interest.

## AUTHOR CONTRIBUTION

**Lina Arcila Hernandez:** Conceptualization (equal); Funding acquisition (supporting); Investigation (equal); Methodology (equal); Project administration (lead); Supervision (equal); Validation (equal); Visualization (lead); Writing‐original draft (lead); Writing‐review & editing (lead). **Kelly Zamudio:** Conceptualization (equal); Funding acquisition (lead); Investigation (equal); Methodology (equal); Project administration (equal); Supervision (equal); Visualization (equal); Writing‐original draft (equal); Writing‐review & editing (equal). **Abby G. Drake:** Conceptualization (equal); Funding acquisition (equal); Investigation (equal); Methodology (equal); Project administration (equal); Resources (equal); Visualization (equal); Writing‐original draft (equal); Writing‐review & editing (equal). **Michelle K. Smith:** Conceptualization (equal); Funding acquisition (supporting); Methodology (equal); Project administration (equal); Visualization (equal); Writing‐original draft (equal); Writing‐review & editing (equal).

## Supporting information

Supplementary Material

## Data Availability

Data sharing not applicable to this article as no datasets were generated or analyzed during the current study.
