# Supplementary material for: Implementing team‐based learning in the life sciences: A case study in an online introductory level evolution and biodiversity course
Source: Ecol Evol. 2020 Dec 17;11(8):3527–36. doi: 10.1002/ece3.6863 (PMC8057328; doi:10.1002/ece3.6863)
Supplement: Supplementary file 1 — Supplementary Material [file ECE3-11-3527-s001.docx]

**Appendix 1**

**Team Survey.** We asked students a combination of the following questions to help us create diverse teams.

- Have you played a team sport in the last 4 years?
- Did you take AP or IB Biology?
- Have you previously taken this course and then dropped it?
- Are you planning to be a BIO major?
- What time zone will you be in while taking this course?
- Is a member of your immediate family a scientist?
- How far is your home from campus? (Options: Less than 3000 miles, More than 3000 miles).
- At what time would you prefer to meet with your team? (Options: Weekday mornings, Weekday afternoons, Weekday evenings, Weekend mornings, Weekend afternoons, Weekend evenings.)
